# MOF-Enhanced Aluminosilicate Ceramic Membranes Using Non-Firing Processes for Pesticide Filtration and Phytochrome Removal

**DOI:** 10.3390/nano14110944

**Published:** 2024-05-27

**Authors:** Liping Zhao, Jinyun Xu, Ming Li, Yanyan Ji, Yu Sun, Ziqi Zhang, Xudong Hu, Zhe Peng, Yicong Wang, Chunming Zheng, Xiaohong Sun

**Affiliations:** 1 Tianjin Key Laboratory of Green Chemical Technology and Process Engineering, State Key Laboratory of Separation Membrane and Membrane Processes, School of Chemical Engineering, Tiangong University, Tianjin 300387, China; 2130030422@tiangong.edu.cn (L.Z.); lming98chem@163.com (M.L.); jiyanyan@tiangong.edu.cn (Y.J.); sunyu@tiangong.edu.cn (Y.S.); 2130030429@tiangong.edu.cn (Z.Z.); 2311530213@tiangong.edu.cn (Z.P.); 2011530208@tiangong.edu.cn (Y.W.); 2 Key Laboratory of Advanced Ceramics and Machining Technology, Ministry of Education, School of Materials Science and Engineering, Tianjin University, Tianjin 300072, China; huxd@tju.edu.cn

**Keywords:** non-firing ceramics, Fe-MOF, adsorption, chlorophyll

## Abstract

Aluminosilicates, abundant and crucial in both natural environments and industry, often involve uncontrollable chemical components when derived from minerals, making further chemical purification and reaction more complicated. This study utilizes pure alumina and fumed silica powders as more controllable sources, enhancing aluminosilicate reactivity through room temperature (non-firing) processing and providing a robust framework that resists mechanical stress and high temperature. By embedding iron-based metal–organic frameworks (Fe-MOF/non-firing aluminosilicate membranes) within the above matrix, these ceramic membranes not only preserve their mechanical robustness but also gain significant chemical functionality, enhancing their capacity to removing phytochromes from the vegetables. Sodium hydroxide and sodium silicate were selected as activators to successfully prepare high-strength, non-firing aluminosilicate membranes. These membranes demonstrated a flexural strength of 8.7 MPa under wet-culture conditions with a molar ratio of Al_2_O_3_:SiO_2_:NaOH:Na_2_SiO_3_ at 1:1:0.49:0.16. The chlorophyll adsorption of spinach conducted on these membranes showed a removal rate exceeding 90% at room temperature and pH = 9, highlighting its potential for the selective adsorption of chlorophyll. This study underscores the potential of MOF-enhanced aluminosilicate ceramic membranes in environmental applications, particularly for agricultural pollution control.

## 1. Introduction

Sintering is a process driven by heat or pressure to compact and form a solid without the powder reaching its melting point [1]. This process is primarily driven by surface energy, which converts the powdered material into a dense body [2]. Changes in microstructure affect the properties of the final product.

High processing temperatures and long sintering times lead to high energy consumption, which leads to economic and environmental problems [3]. These challenges have led to the search for alternative low-temperature processing methods. In order to reduce the sintering temperature of oxide ceramics and thus the associated energy consumption, researchers have explored various strategies. For example, alumina ceramics are prepared by synthesizing alumina powders by non-combustion methods, by reducing the sintering temperature, by ice template method, or by doping ceramic composites to reduce the sintering temperature of alumina ceramics [4,5,6,7]. Other methods include ball milling to refine powders [8,9], microwave and plasma sintering [10], 3-D printing [11,12,13,14], etc. These methods indicate a trend towards increasing energy efficiency in production. They also help to reduce greenhouse gas emissions, aligning with global efforts to minimize the carbon footprint of industrial processes.

Non-firing or room temperature processing ceramics have gained significant attention for their unique advantages in manufacturing and energy efficiency, particularly for environmental remediation applications [15,16]. Unlike conventional sintering processes, non-firing ceramics can be formed at much lower temperatures, allowing for significant energy savings and a reduction in carbon emissions [17]. This innovative sintering method uses mechanical or chemical treatments to activate particle surfaces, making them highly reactive and capable of forming strong bonds without high-temperature firing [18,19,20,21,22].

The structural integrity of aluminosilicate ceramics, achieved through non-firing processes, provides a robust framework that resists mechanical stress and high temperatures [17,23]. This durability is crucial for industrial applications, where materials must endure harsh conditions [24,25]. By embedding Fe-MOFs (iron-based metal–organic frameworks) within this matrix, these composites not only preserve their mechanical robustness but also gain significant chemical functionality [26,27]. MOFs, known for their high surface area and porosity, introduce dynamic adsorptive capabilities [28,29,30], making these ceramic membranes highly effective at capturing and removing organic pollutants from various environments, including agricultural settings where vegetable juice purification is critical [31,32].

In their study of ceramic-MOF, Sun et al. [33] improved the performance of ceramic-based permeation membranes by incorporating in situ-grown Zr-MOF as an intercalation layer, achieving robust water treatment performance under harsh conditions and the efficient separation of industrial petrochemical wastewater. Usman et al. [34] used the solvothermal growth of UiO-66-NH_2_ MOF in situ on polydopamine (PDA)-functionalized ceramic membranes (CM), and finally prepared a defect-free hydrolytically stable zirconium-based MOF separation layer by a two-step method. PDA-s-UiO-66-NH_2_-CM membrane exhibits superhydrophilic properties in air and superoleophobicity underwater, with extremely high permeability and oil–water separation ability. Torrez-Herrera et al. [35] synthesized a ceramic-MOF filter using an aluminum salt slag fertilizer, and used the prepared ceramic-MOF filter to investigate the adsorption of CO_2_. Ceramics and MOF composites have been researched extensively [36,37,38], but the application of non-firing ceramic-MOF membrane materials for the adsorption of plant pigments is relatively rare.

The integration of Fe-MOFs into the aluminosilicate matrix (Fe-MOF/non-firing aluminosilicate membranes) transforms these ceramics into advanced adsorptive membrane materials that can effectively remove pesticides, chlorophyll, and other contaminants from vegetable juices [39]. This capability is particularly important for ensuring the safety and quality of agricultural products. Unlike traditional methods, which might use activated carbon or chemical treatments that can alter taste or leave residues, the Fe-MOF/non-firing aluminosilicate membranes offers a non-invasive and residue-free option. It selectively absorbs contaminants without affecting the essential qualities of the juice. Additionally, the thermal stability of the membranes ensures that it can withstand the pasteurization temperatures commonly used in juice processing, adding an extra layer of convenience and safety.

One of the standout features of non-firing aluminosilicate ceramics, especially those integrated with Fe-MOFs, is their reusability and high separation efficiency. This reusability not only makes them cost-effective but also environmentally friendly, as it reduces the need for frequent replacement and minimizes waste [40,41]. The high separation efficiency ensures that even trace amounts of contaminants are effectively removed, enhancing the purity of the final product. This aspect is crucial in applications where maximum residue limits (MRLs) are strictly regulated, such as in the food and beverage industry [42,43].

In practical applications, the Fe-MOF/non-firing aluminosilicate membranes have demonstrated promising results in removing phytochromes like chlorophyll from vegetable juices. The optimized pore structure and properties of the membranes enhance their efficiency by enabling selective adsorption, which minimizes undesirable interactions and maximizes the removal rate [44]. These capabilities not only improve the purity of the final product but also support compliance with good agricultural practices by reducing the levels of environmental pollutants in food products.

The development and application of Fe-MOF/non-firing aluminosilicate membrane enhancements contribute positively to sustainability. By lowering energy requirements and CO_2_ emissions compared to traditional methods, and by improving food safety through effective pollutant removal, these ceramic membranes support broader efforts to mitigate environmental impact [45,46]. They represent a forward-thinking solution to the challenges of modern agricultural and environmental remediation [47].

Fe-MOF/non-firing aluminosilicate membranes mark a significant advancement in material science, particularly in the context of environmental remediation and agricultural applications. By combining mechanical durability with superior chemical functionality, and by offering reusable, high-efficiency contaminant separation, the membranes could provide a sustainable, cost-effective alternative to the conventional materials and methods used in juice processing and other related industries. The research undertaken in this experiment fills a gap in the application of ceramic-MOF membrane materials in plant pigment adsorption. This research and development result could find a new path forward in the growing trend towards sustainable industrial practices and ongoing innovation in materials science aiming at enhancing ecological and food safety standards.

## 2. Materials and Methods

### 2.1. Reagents

α-Alumina (99.99%) comes from Huawei Ruike Chemical Co., Ltd. (Beijing, China) with a particle size of 500 nm. The brand of silica (99.8%) is Degussa, Germany, and the model is AEROSIL 200, with a specific surface area of 200 m^2^/g, hydrophilic type. The waterglass and powder sodium silicate are from Yousuo Chemical Technology Co., Ltd. (Shandong, China). Acetonitrile and anhydrous magnesium sulfate are from Fengchuan Chemical Reagent Technology Co., Ltd. (Tianjin, China). FeCl_3_∙6H_2_O was purchased from Sanjiang Serida Trading Co., Ltd. (Tianjin, China). 1,3,5-Benzenetricarboxylic acid was purchased from Dibai Biotechnology Co., Ltd. (Shanghai, China). Sodium acetate anhydrous was sourced from Macklin’s Biochemical Technology Co., Ltd. (Shanghai, China). Acetic acid was purchased from Aladdin Biochemical Technology Co., Ltd. (Shanghai, China). NaOH was purchased from Tengzhun Biotechnology Co., Ltd. (Shanghai, China). Polyvinylpyrrolidone was purchased from Maidin Technology Co., Ltd. (Tianjin, China). All above reagents are analytically pure. The pesticide is imidacloprid (70% active ingredient). Deionized water was used throughout the work.

### 2.2. Instrumentation and Analytical Conditions

Powder X-ray diffraction (XRD) spectra were measured using Bruker D8 Advance, Germany. Strength tests were measured on a DR-507 universal testing machine using sheet parameters with sample strip parameters of 2 mm × 3 mm × 22 mm. A Nicolet iS50 Fourier Transform Infrared (FT-IR) spectrometer from Thermo-Fisher, Horsham, UK, was used. Nitrogen adsorption and desorption tests were performed using a Mack ASAP 2460 model. The surface morphology and particle size of the samples were observed by scanning electron microscopy (SEM) using a Hitachi S4800 model (HITACHI, Tokyo, Japan). We performed comprehensive thermal (TG-DSC) analysis with a simultaneous thermal analyzer model STA449 F5 from NETZSCH Manufacturing GmbH, Frankfurt, Germany, with a heating rate of 10 °C/min in the range of 50 °C–800 °C. The main instruments used in the experiment were a planetary ball mill (Qidong Honghong Instrument Equipment Factory, Qidong, China, KE-0.4L), precision diamond wire cutting machine (Shenyang Kejing Automation Equipment Co., Ltd., SXT-202AQ, Shenyang, China), density meter (Dongguan Dongri Instrument Co., Ltd., DR-200A, Dongguan, China), and an ultraviolet spectrophotometer (Shanghai Jinghua Technology Instrument Co., Ltd., 754PC, Shanghai, China).

### 2.3. Preparation of Non-Firing Aluminosilicate Ceramic Membranes

First, alumina and silica powders were mixed at a molar ratio of 1:1 by mechanical ball milling at 300 rpm for 60 min, with zirconia balls of 5 mm in size and a ball-to-powder ratio of 10:1. The ball mill powder not only performs the homogeneous mixing of the powder, but also activates the surface of the powder, making the reaction required for producing oxide ceramics easier. The mixed powder was processed through a 60-mesh sieve to prevent powder agglomeration during the ball milling process. If the sample contained powder sodium silicate, the powder sodium silicate was mixed with the powder in proportion to the ball mill to ensure the uniformity of the powder.

Sodium hydroxide was dissolved in deionized water, and a sodium activator of pure sodium hydroxide was prepared. Waterglass and powder sodium silicate were added to prepare an activator containing sodium silicate. All alkali activators were then ready for use. The powder, after ball milling, was mixed with alkali activator and stirred for 10 min, and then poured into a mold (ϕ25 mm * 8 mm) to cure and dry in a thermal and humidity test chamber at different times and in different modes. The main factors affecting the properties of ceramic membranes were explored, aiming to obtain the best ratio.

### 2.4. Preparation of Fe-MOF

Fe-MOF samples were synthesized by an improved method according to the literature [48]. The main steps were as follows: 0.015 mol FeCl_3_∙6H_2_O (4.05 g) was taken in a polytetrafluoroethylene (PTFE) container, 75 mL of ultrapure water was added to dissolve it, and 0.01 mol benzene tricarboxylic acid (BTC, 2.10 g) was added; the PTFE container was put into a hydrothermal synthesis reactor and put into an electric thermostatic drying oven and synthesized for 24 h at 150 °C; the product was centrifuged and washed with ultrapure water three times until the supernatant was colorless and clarified, then soaked in anhydrous ethanol for 4 h and centrifuged two times to obtain the precipitate, and then dried at 80 °C in an electric thermostatic drying oven to obtain the Fe-MOF samples.

### 2.5. Preparation of Fe-MOF/Non-Firing Aluminosilicate Membranes

The process begins with the preparation of sodium-excited aluminosilicate ceramic membranes, which are not subjected to a sintering process to maintain their porous structure. These ceramics were first shaped into sheets with a thickness of approximately 2 ± 0.5 mm using a precision diamond wire cutting machine, ensuring uniform thickness across samples for consistent experimental conditions. The slurry for the substrate coating was prepared by mixing metal–organic frameworks (MOFs) and polyvinylpyrrolidone (PVP) in a solvent mixture of ethanol and water at a mass ratio of 3:1. This homogeneous mixture was then evenly coated on the surface of the aluminosilicate substrates to a controlled thickness ranging from 1 to 2.5 mm. The specifics of the slurry ratios and the detailed composition related to aluminosilicate ceramic membranes are tabulated (refer to Table 1), highlighting variations in molar ratios of NaOH/SiO_2_, which are crucial for tailoring the physical properties of the ceramic membranes. After coating, the substrates were air-dried at room temperature to evaporate the solvents effectively, ensuring the formation of a sturdy MOF–PVP composite film on the membrane substrates. The curing of the substrates was performed under wet conditions at 60 °C, which facilitates the formation of a robust composite structure by enhancing the interaction between the ceramic membrane surface and the MOF–PVP coating.

### 2.6. Application of Fe-MOF/Non-Firing Aluminosilicate Membranes for Phytochromes Removal

The change in chlorophyll concentration was used to calculate the removal efficiency of phytochromes by alkali-excited prepared Fe-MOF/non-firing aluminosilicate membranes. The calculation method is consistent with that previously reported in the literature [44]. The chlorophyll adsorption experiments were carried out on samples with different sodium hydroxide contents, different temperatures, different pH levels, different solution amounts, and different coating thicknesses. The absorbance test was carried out in the wavelength range of 200–800 nm using a UV spectrophotometer. Chlorophyll has a strong absorption band at 630–670 nm in the red-light absorption band. The absorption peak of chlorophyll a was at 645 nm and that of chlorophyll b was at 663 nm. The chlorophyll concentration (C_c_) and the removal rate of chlorophyll concentration (R%) were calculated as follows:

Chlorophyll concentration (C_c_):

C_c_ = 8.05 × A_663_ + 20.29 × A_645_

Chlorophyll removal (R%):

$$R(\%)=\frac{C_{0}-C_{e}}{C_{0}}\text{×}100\%$$

where

A_663_: absorbance of the solution at 663 nm;
A_645_: absorbance of the solution at 645 nm;
C_0_: initial concentration of phytochromes (mg/L);
C_e_: remaining concentration of phytochromes (mg/L).

Following the official method of the AOAC, phytochromes were extracted from vegetables [49]. The phytochromes in the extract were composed of chlorophyll a, chlorophyll b, chlorophyll c, lutein, carotene, etc. We used the change in chlorophyll concentration to calculate the removal efficiency of the prepared composites for phytochromes. Homogenized spinach juice was prepared using 10.0 g of fresh spinach leaves after the removal of surface dust and 200 mL of deionized water. The larger cellulose and impurity particles were removed from the spinach juice using a sieve. Another 40 mL of deionized water was added to 5 mL of spinach juice, which was the stock solution of chlorophyll used in the experiment, in order to make the removal effect more obvious. The treated filtrate was mixed with 10 mL of acetonitrile (containing 1% acetic acid), and the mixture was vortexed in a vortex mixer at 1200 rpm/min for 5 min. 1.0 g of sodium acetate anhydrous and 4.0 g of anhydrous magnesium sulfate were added and the mixture was immediately vortexed for another 2 min. Finally, the organic phase was centrifuged at 9000 rpm for 5 min. The UV-Vis absorption spectra were scanned in the wavelength range of 200–800 nm using the phytochromes solution (organic phase) to obtain the full absorption spectra.

## 3. Results and Discussion

### 3.1. Preparation of Fe-MOF/Non-Firing Aluminosilicate Membranes and Adsorption Performance Testing

The prepared substrates, designated as samples Y1, Y2, and Y3, exhibited varying physical properties as detailed in the Material Data section (Table 1). Due to the dense nature of the Y4 sample and its low specific surface area and unclear pore size distribution, it is not suitable for use as adsorbent membrane. Therefore, the final substrate membranes were Y1, Y2, and Y3. MOF and PVP were homogeneously mixed in a solution of ethanol and water at a mass ratio of 3:1, and then the homogeneous slurry was coated on the surface of the substrate with a thickness of 1–2.5 mm, and then dried at room temperature. After drying, the Fe-MOF/non-firing aluminosilicate membranes sample was obtained.

**Scheme 1:**
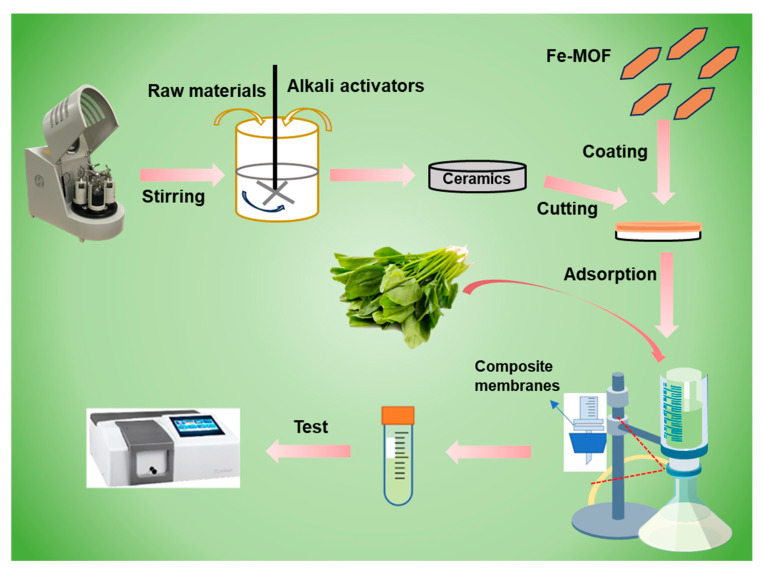
Preparation of Fe-MOF/non-firing aluminosilicate membranes and their application in vegetable sample pretreatment.

The preparation of Fe-MOF/non-firing aluminosilicate membranes and their application in vegetable sample pretreatment are shown in Scheme 1. Traditional ceramic sintering processes require high temperatures, which significantly contribute to energy consumption and carbon emissions. This low-temperature process does not compromise the structural properties of the membranes, which is critical for maintaining the durability required for industrial applications. By embedding Fe-MOFs within the aluminosilicate matrix, the membranes gain enhanced adsorptive properties due to the high surface area and porosity of the Fe-MOFs. This integration enables the effective trapping and adsorption of organic chlorophyll from vegetable juices without altering their natural flavor or leaving harmful residues. The specific interaction between Fe-MOFs and contaminants leads to high removal efficiencies, making these composites ideal for applications. 

### 3.2. Characterization of Microscopic Morphology and Material Composition

**Figure 1:**
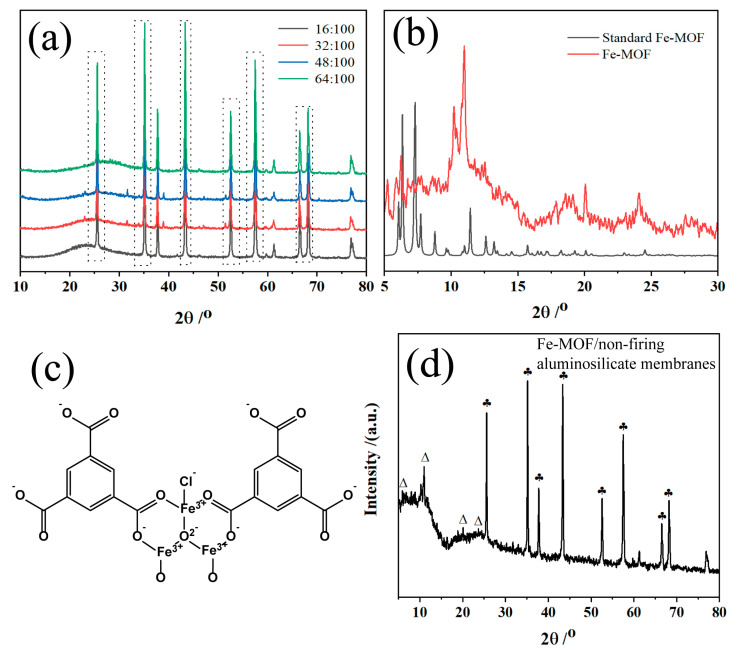
(**a**) XRD of aluminum silicate non-firing ceramic membranes at molar ratios of NaOH/SiO_2_ of 16:100, 32:100, 48:100, and 64:100; (**b**) XRD of Fe-MOF synthesized by hydrothermal method; (**c**) Structural formula of Fe-MOF synthesized by hydrothermal method; (**d**) XRD of Fe-MOF/non-firing aluminosilicate membranes: ♣ alumina (PDF#99-0036), ∆ Fe-MOF.

The XRD patterns in Figure 1a represent the aluminosilicate non-firing membranes synthesized with different concentrations of sodium hydroxide. All the XRD peaks for these membranes are sharp alumina peaks, which indicates the crystalline phase of alumina, showing the dominance of alumina in the ceramic membrane composition. The 2θ peaks around 15–30° imply the existence of amorphous phases within the membrane matrix, likely due to the presence of amorphous aluminosilicates. With the addition of sodium hydroxide, the intensity of the 23.1, 31.5, 39.1, and 51.5° peaks increased with the sodium hydroxide content. These patterns suggest that the higher concentrations of sodium hydroxide facilitate the chemical reaction between the alkali and the aluminosilicate particles, potentially leading to the formation of additional crystalline phases. XRD peaks are critical in determining the amorphous phases of aluminosilicates because they are directly related to the internal structure of the formed ceramic membrane. The changes observed in the XRD patterns with varying sodium hydroxide content highlight the role of the alkali activator in the optimization of the properties of ceramic membranes. As the sodium hydroxide content increases, there is likely a more extensive dissolution of the aluminosilicate precursors, followed by reorganization and polycondensation to form a three-dimensional geopolymeric network. This network is responsible for the mechanical strength and durability of the ceramic membranes.

Moreover, XRD analysis provides insight into the optimization of the non-firing ceramic membranes producing process. By controlling the amount of sodium hydroxide, the properties of the ceramic membranes can be adjusted to improve the mechanical properties and chemical resistance required for the adsorption of phytochromes. The above results show the balance between the reactants in the synthesis of aluminosilicate-based non-firing ceramic membranes and the importance of precise control over synthesis conditions in order to achieve the desired product characteristics.

The XRD patterns of Figure 1b show the successful synthesis of Fe-MOFs prepared via hydrothermal method. In Figure 1b, the Fe-MOF material demonstrates the peak positions consistent with the work of Song and Guesh, et al. [48,50], and the main outgoing peaks are within 2θ of 15°. Since the raw materials for the synthesis of Fe-MOF using the hydrothermal method are FeCl_3_∙6H_2_O and benzene tricarboxylic acid, and since it is known that Fe-MOF was successfully prepared according to Figure 1b, it is inferred that the structural formula of Fe-MOF generated in this experiment is shown in Figure 1c. Meanwhile, Figure 1d reveals the crystalline structure of aluminosilicate-based membranes, predominantly characterized by alumina phases and interspersed with Fe-MOF peaks. The retention of Fe-MOF peaks shows that the composites benefit from the combined properties of Fe-MOF and the aluminosilicate matrix. While the Fe-MOF contributes its potential adsorptive properties, the matrix likely enhances the mechanical strength and thermal stability of the membranes. The resulting membranes could exhibit improved adsorption performance with the distinctive properties of Fe-MOF and the robustness provided by the aluminosilicate framework.

**Figure 2:**
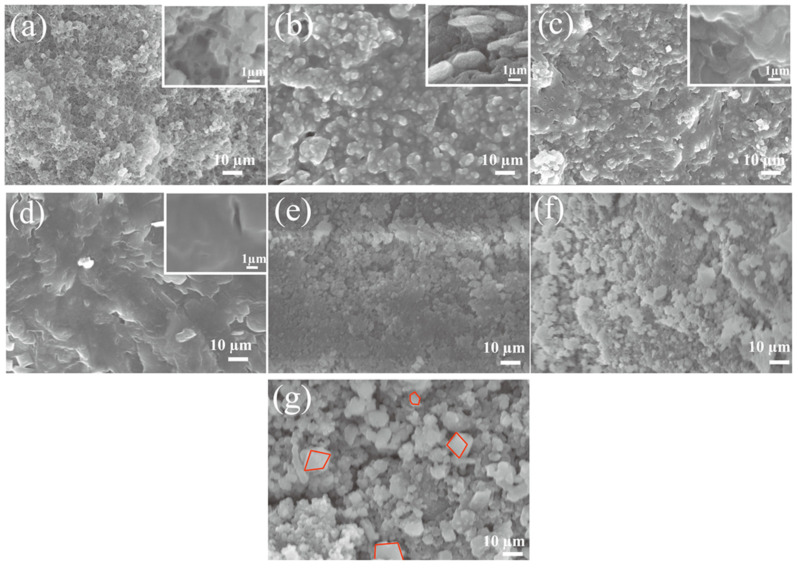
SEM of aluminum silicate non-firing ceramics with molar ratios of sodium hydroxide/silica of 16:100 (**a**), 32:100 (**b**), 48:100 (**c**), and 64:100 (**d**), respectively; (**e**) SEM of aluminum silicate ceramics with only NaOH added; (**f**) Aluminum silicate ceramics with sodium hydroxide and Na-waterglass added; (**g**) SEM of the Fe-MOF/non-firing aluminosilicate membranes.

Figure 2a–d shows SEM images of aluminum silicate ceramic membranes prepared with different sodium hydroxide/silica molar ratios (NaOH/SiO_2_), showing the microstructural changes in aluminum silicate non-firing ceramic membranes with increasing sodium hydroxide content. With the lowest NaOH/SiO_2_ molar ratio of 16:100, the ceramic membrane shows a substantial presence of unreacted particles and a porous structure, indicating the geopolymeric reaction is incomplete. As the NaOH molar ratio rises to 32:100, there is a marked increase in aluminosilicate derivatives, suggesting a more extensive chemical interaction facilitated by the NaOH [51]. With a 48:100 ratio of NaOH, the image reveals aluminosilicate polymers, signaling a transition towards a more cohesive and less porous structure, indicative of a further advanced reaction stage. The progression culminates in Figure 2d at a concentration of 64:100, showing a dense gel-like morphology with significantly reduced porosity, representing the better geopolymerization process at a high NaOH concentration. This trend shows the crucial role of NaOH in transforming the aluminosilicate matrix, as higher concentrations lead to a more complete reaction, producing a dense ceramic with improved mechanical strength and durability. The SEM images show the essence of the geopolymerization process, which is fundamentally governed by the amount of NaOH, which influences the final material properties and performance of the ceramic membranes. Figure 2e shows the SEM image of the sample membrane prepared by adding only sodium hydroxide; it illustrates that the addition of sodium hydroxide causes a reaction between the raw materials, which makes the particles pile up on each other, but there are a large number of unreacted particles in the sample. Figure 2f shows SEM images of an aluminosilicate membrane material with sodium hydroxide and Na-silicate additions. As can be seen from the figure, the introduction of a certain amount of aluminosilicate makes the reaction of the membrane more complete, and more aluminosilicate is generated; however, the sample membrane is still relatively dense, and if it is used for liquid filtration, there will be certain difficulties. Figure 2g is the SEM image of the Fe-MOF/non-firing aluminosilicate membranes. The MOF crystal can be clearly observed from the figure (red area), indicating that the composite method is feasible. The cubic structure of the MOF crystal on the surface of the membrane [52] is obvious, and the pore structure of the membrane itself is not affected, and the pores are abundant for this membrane.

**Figure 3:**
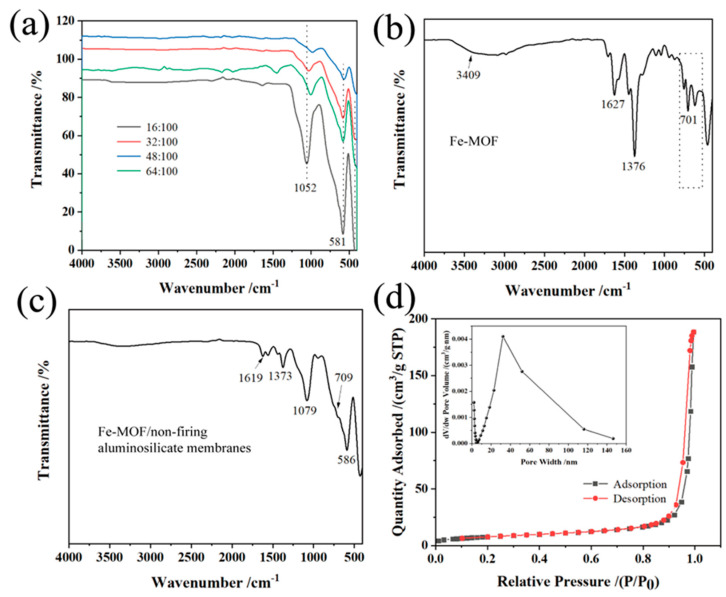
(**a**) FT-IR of aluminum silicate ceramics prepared with different sodium hydroxide/silica molar ratios; (**b**) FT-IR of Fe-MOF prepared by hydrothermal method; (**c**) FT-IR of Fe-MOF/non-firing aluminosilicate membranes; (**d**) Nitrogen adsorption–desorption curves and particle sizes of aluminum silicate ceramics with sodium hydroxide/molar ratio of 16:100.

The FT-IR spectra in Figure 3a show the properties of non-firing aluminosilicate ceramic membranes synthesized with different sodium hydroxide concentrations. Peaks at 1619 cm^−1^ indicate the hydroxyl group vibrations of ceramics structure. Absorptions at 1079 and 1052 cm^−1^ correspond to the asymmetric vibrations of the Si-O-Al and Si-O-Si bonds within the ceramics [53,54], showing the primary bands formed during geopolymerization. The bands between 695 and 717 cm^−1^ and 560 and 590 cm^−1^, along with 460–475 cm^−1^, are attributed to the stretching of Al-O bonds and the symmetric stretching and bending vibrations of Si-O-Al and Si-O-Si bonds, respectively [55,56]. The intensity of bands in the 1000–1100 cm^−1^ range is directly related to the anticipated compressive strength; a higher intensity suggests a greater mechanical strength [57].

Figure 3b shows the FT-IR spectrum of Fe-MOF synthesized via hydrothermal method. According to previous literature [58], Fe-MOF consists of benzene carboxylates; therefore, the characteristic IR spectrum of Fe-MOF mainly reflects the benzene carboxylate. The bands at 1627 cm^−1^ are attributed to the C=O bond in the carboxylate, respectively. The band at 1376 cm^−1^ arises from aromatic carbon C-C vibration modes, while peaks around 540–750 cm^−1^ align with Fe-OH groups, indicating the inclusion of iron within the organic framework.

From Figure 3c, the FT-IR spectra show the combined results of the base aluminosilicate membrane and the Fe-MOFs without the emergence of new phases. This suggests a physical integration of components rather than a chemical transformation, confirmed by observations from X-ray diffraction studies. The absence of new phase formations indicates a mixture rather than a reaction between the aluminosilicate base and Fe-MOF, pointing to the composite nature of the membrane material.

Overall, the FT-IR analysis is a powerful tool for probing structural and chemical interactions within aluminosilicate-based ceramic membranes and their composites. The spectra detail how the presence of various activators and the modification of sodium hydroxide content influence the formation and properties of these membranes. Such insights are critical for tailoring the mechanical strength and durability of ceramic membranes for specific industrial applications.

Figure 3d shows the N_2_ adsorption–desorption curve and particle size analysis of the aluminosilicate non-firing ceramic membranes. The curve shows an H3-type hysteresis, indicating uneven porosity, such as the cracks that are prevalent in mixed porosity materials [59]. The pore size distribution further shows that the aluminum silicate membrane has a predominantly mesoporous and macroporous feature [60] when NaOH is added at a low amount, which confirms that its porosity is beneficial for applications requiring high surface area. For example, as shown in Table 1, the BET surface area is the largest at 28.2 m^2^/g at the lowest NaOH concentration, indicating a large number of reaction sites. The nitrogen adsorption–desorption curves of the aluminum silicate ceramic membranes for other sodium hydroxide/silica molar ratios are shown in Figure S1a,b. As the sodium hydroxide/silica molar ratio increases, the prepared aluminosilicate samples become more and more dense, especially at the ratio of 64:100, indicating the non-mesoporous structure of the membranes.

**Figure 4:**
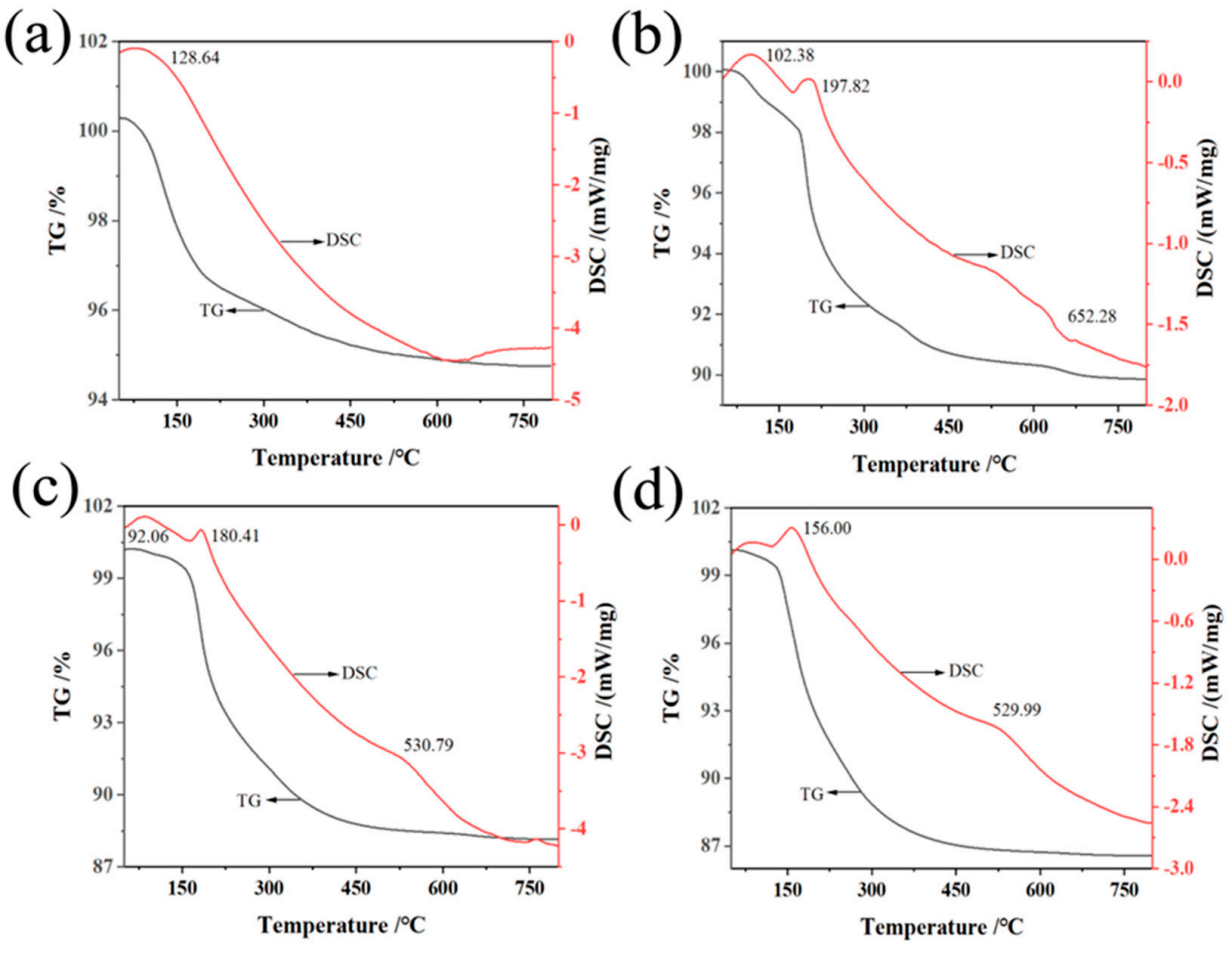
TG-DSC of non-firing aluminum silicates with different sodium hydroxide/silica molar ratios: (**a**) 16:100, (**b**) 32:100, (**c**) 48:100, (**d**) 64:100.

The thermogravimetric (TG) analysis and differential scanning calorimetry (DSC) of aluminosilicate non-firing ceramic membranes in Figure 4 reveal a critical dependence of phase transformations on temperature, influenced by the content of sodium hydroxide. As the NaOH concentration rises from 16:100 to 64:100, the temperature at which the endothermic peaks occur shifts leftward, indicating that the phase transformations begin at lower temperatures with higher NaOH content. This is evidenced by a single weight loss stage for the samples with 16:100 and 64:100, and three distinct stages for those with 32:100 and 48:100. The first weight loss under 100 °C represents the loss of free water, while the next loss up to 200 °C is due to bound water escaping [61]. Most notably, the weight loss observed between 500–650 °C is a result of the breakdown of the aluminosilicate gel, a key structural component. The decomposition of the gel indicates a significant phase transition in the ceramic membrane, with more NaOH leading to an increase in hydration products and a more pronounced gel formation, thus altering the membranes’ phase structure. Concurrently, a reduced rate of initial weight loss at higher NaOH concentrations suggests less free water, reflecting a denser material phase with decreased porosity. The aggregate weight loss percentages rise with increasing NaOH, suggesting that the amount of water involved in the hydration products within the membranes’ structure becomes more substantial, confirming a material that is rich in aluminosilicate gel.

These TG-DSC curves are not just temperature records; they map the evolution of the phase states of membranes with temperature, which is crucial for applications requiring precise thermal control. For instance, the increased hydration product at higher temperatures with more NaOH indicates a ceramic membrane phase that is thermally less stable but may have enhanced reactivity for certain chemical processes. This nuanced understanding of phase changes relative to temperature and NaOH content is vital for developing ceramics tailored for high-temperature applications or for those requiring specific thermal behaviors.

### 3.3. Characterization of Mechanical Properties

**Figure 5:**
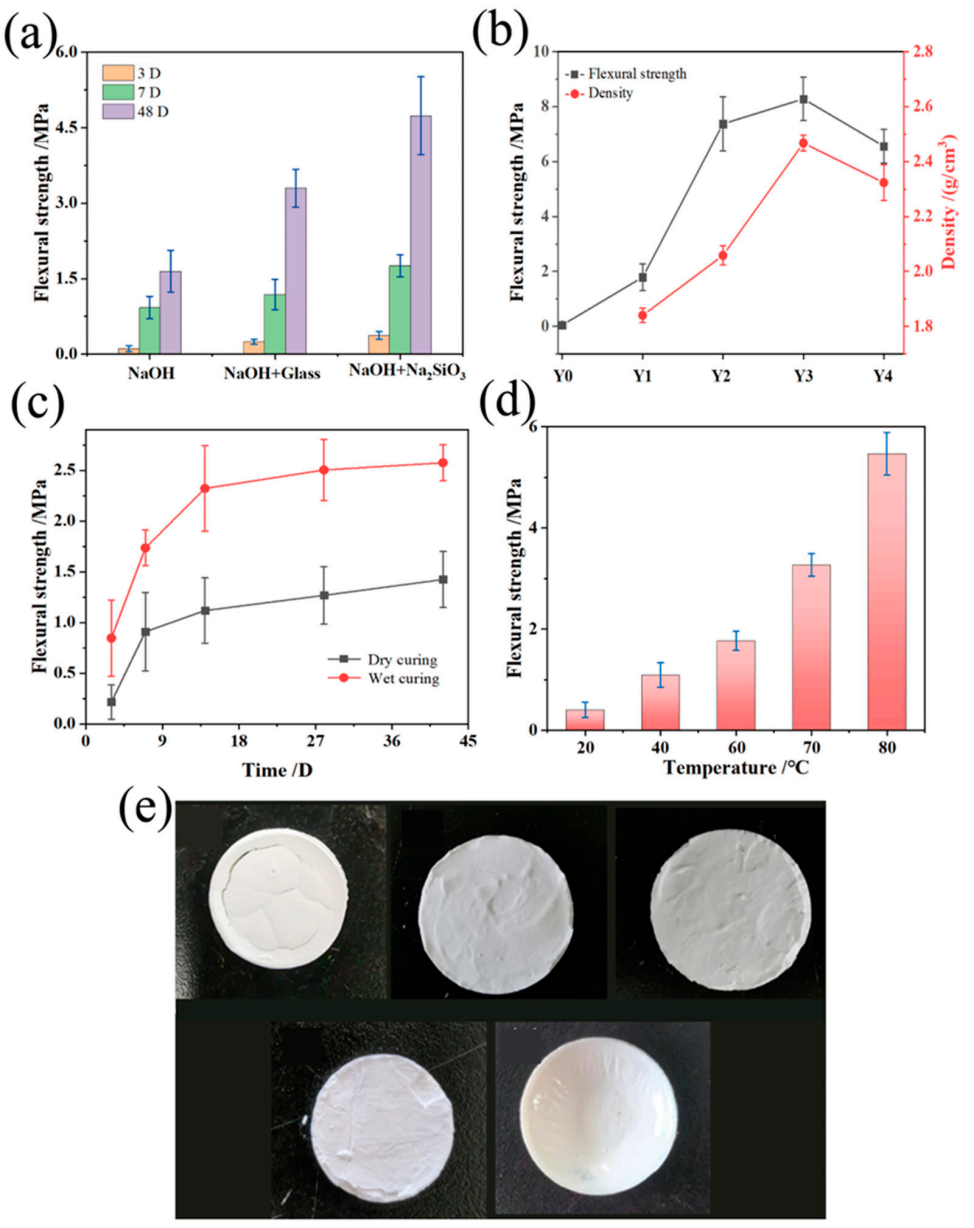
Non-firing aluminosilicates. (**a**) Flexural strength for different sodium silicate types; (**b**) Flexural strength for different sodium hydroxide contents; (**c**) Flexural strength for different curing types; (**d**) Flexural strength for different curing temperatures and (**e**) physical drawings.

The macroscopic mechanical properties of aluminosilicate non-firing ceramic membranes, particularly flexural strength, are crucial for their application performance. This strength is inherently dependent on the type of alkali activator, mix duration, curing environment, and the proportion of sodium hydroxide used in the synthesis. In the strength test, six specimens were taken from each parallel experiment and the average value was calculated to derive the fracture load of the specimen. As shown in Figure 5a, the use of sodium hydroxide in conjunction with waterglass and powdered sodium silicate as alkali activators significantly enhances flexural strength, with an optimum observed at a 48-day maturation, indicating a favorable reaction progression and structural integrity. Specifically, samples prepared with a sodium hydroxide and powdered sodium silicate blend demonstrate superior flexural strength, attributable to the additional aluminosilicate content increasing alkalinity and promoting the formation of a robust aluminosilicate matrix.

Figure 5b shows the flexural strength and density of non-firing aluminosilicate ceramic membranes prepared at different sodium hydroxide concentrations. The sodium hydroxide content plays a dual role. It enhances the dissolution of silicon and aluminum, thus increasing the flexural strength, while excessive amounts may lead to ion pair effects that hinder gel growth and result in “passivation” of the Si-O and Al-O structures. Such passivation hampers chemical bonding within the aluminosilicate gel matrix, causing a downturn in strength. The samples with high NaOH content displayed a softer texture under compression, suggesting an abundance of un-dried gel material, which undermines the realizable strength in a given testing period.

This is shown in Figure 5c: curing conditions further modulate flexural strength, with sealed, moist environments ensuring consistent moisture levels, facilitating the maturation of strength over time. In contrast, dry curing can lead to the rapid evaporation of water, causing sample shrinkage, surface cracking, and a resultant reduction in strength. A 42-day wet-cured sample showcased a flexural strength of 2.5 MPa, significantly higher than its dry-cured counterpart, reinforcing the need for controlled humidity during curing. 

The physical macroscopic image of aluminosilicate non-firing ceramic membranes post-calcination projected in Figure 5e depicts the impact of the curing temperature on the membranes’ structural integrity and consequent mechanical performance. This relationship is further elucidated by the flexural strength measurements displayed in Figure 5d, showcasing the critical role that temperature plays in the reaction kinetics and product formation of the ceramic membranes. The difference in time and temperature to flexural strength was quite significant after Student’s *t*-test (*p* < 0.05 and 0.003). 

At lower temperatures, membranes fail to dry completely within the test period, resulting in cracks and compromised integrity upon subsequent air drying. As the curing temperature increases, the membranes’ integrity improves, with flexural strength progressively rising. This strength increment is particularly significant between 60 °C and 80 °C, jumping by 208% to reach 5.4 MPa. Such enhancement indicates more complete chemical reactions at higher temperatures. Increased curing temperature accelerates the reaction rate of the liquid activator with solid precursors and expedites the growth of the colloidal network. Consequently, there is a reduction in macroporosity and an increase in microporosity, yielding a denser colloidal structure and thus heightened flexural strength. Nonetheless, curing at temperatures above 80 °C results in rapid water loss, leading to high crack rates in the samples. Water content during slurry preparation also influences flexural strength, but only to a minor degree within the range of water volumes investigated. The addition of water facilitates the dissolution of powders in the sodium hydroxide solution, enhancing the possibility of the aluminosilicate reaction, producing more uniform slurries with fewer air bubbles; however, excessive water reduces the reaction rate, prolongs the curing and drying time, and ultimately affects the flexural strength of the membranes. In essence, the above results suggest the flexural strength of aluminosilicate non-firing ceramic membranes is finely tuned by the sodium hydroxide content, curing temperature, and curing conditions. These factors synergistically determine the final mechanical attributes, dictating the ceramic membranes’ performance within their intended reaction systems. 

Figure S1c,d shows the effect of different curing times and water contents on the strength of ceramic membranes. From the figures, it can be seen that the curing time has an effect on the flexural strength of the alkali-excited prepared aluminosilicate non-firing ceramic membranes. The flexural strength increases gradually and almost linearly with the increase of the curing time. It may be that the slurry has a certain mobility in a short period of time, and the ions can move freely inside, and with the extension of time, the moisture gradually decreases, and the reaction gradually generates a colloid, which hinders the movement of the ions, and the rate slows down although the strength increases. It can also be seen that in a certain time, the internal reaction of the sample is still continuing. The water content does not have a significant effect on bending strength, which increases slightly as the water content increases. An appropriate increase in water content allows the powder to dissolve more fully in the sodium hydroxide solution, with a greater likelihood of an aluminosilicate reaction occurring, producing a slurry that is more uniform and contains fewer air bubbles. Excessive water content reduces the rate of aluminosilicate reaction and prolongs the time required for conditioning and drying, thereby affecting the flexural strength of the membranes. The test was also statistically significant with Student’s *t*-test (*p* < 0.05).

In sum, the anticipated physical representation of the ceramic membranes highlights the critical influence of curing temperature and water content on the reaction mechanism of the aluminosilicate formation, directly correlating with the macroscopic mechanical properties of the ceramic membranes. This intricate balance of synthesis parameters is paramount for tailoring the material’s final mechanical traits, ensuring its performance aligns with the demands of its intended reaction system.

### 3.4. Characterization of Adsorption Properties

**Figure 6:**
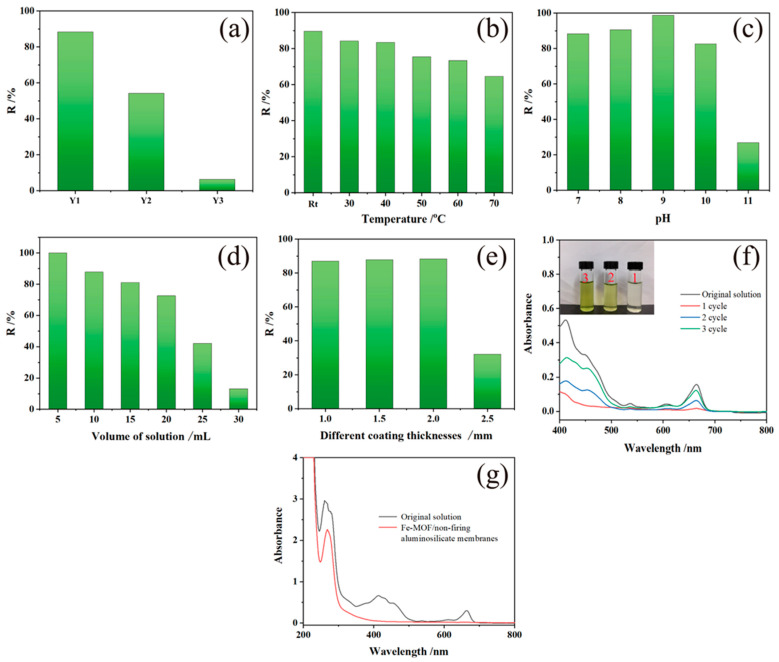
UV-visible absorption spectra and photomicrographs of (**a**) Substrate membranes with different sodium hydroxide contents; (**b**) Different temperatures; (**c**) Different pH levels; (**d**) Different solution amounts; (**e**) Different coating thicknesses; (**f**) UV-visible absorption spectra and photomicrographs of cyclically adsorbed chlorophyll solutions; (**g**) UV-visible absorption spectra of imidacloprid chlorophyll solutions adsorbed with a concentration of 10 ppm.

Figure 6 illustrates the impact of various experimental conditions on the degradation performance of Fe-MOF/non-firing aluminosilicate membranes for chlorophyll adsorption. The experiments investigate the effects of temperature, NaOH content, solution volume, and pH level on the adsorption efficiency.

The relationship between NaOH content and adsorption effectiveness is depicted in Figure 6a. The optimal NaOH addition of 16:100 leads to the highest chlorophyll removal rate and the effectiveness drops drastically with higher NaOH concentrations, aligning with the theory that greater surface area and porosity favor adsorption. Temperature variations reveal that the adsorption capacity is highest at room temperature with a significant decrease in performance at elevated temperatures, as the state of the chlorophyll within the vegetable juice is altered, diminishing the adsorption quality (Figure 6b). Moreover, the pH study (Figure 6c) shows that adsorption rates improve as pH shifts from neutral to slightly alkaline, peaking at pH = 9, and then sharply falling off at pH = 11, possibly due to deprotonation effects at high pH levels, which disrupt the interaction between the Fe-MOF/non-firing aluminosilicate membranes and chlorophyll. 

These findings highlight the sensitivity of the Fe-MOF/non-firing aluminosilicate membranes’ degradation capabilities to environmental conditions. Optimal chlorophyll removal is achieved under room temperature, with moderate NaOH addition, lower solution volumes, and a slightly alkaline pH, emphasizing the need for precise control over these parameters in applications such as water purification, where efficient selective adsorption of organic pollutants is crucial. This research not only informs the operational parameters for using these membranes but also opens pathways for further optimization in similar environmental remediation technologies.

Lastly, as shown in Figure 6d, adsorption efficiency tends to decrease with increasing solution volume, suggesting a saturation of the adsorption sites or a dilution effect.

Figure 6e addresses the role of MOF coating thickness in the adsorption process. The optimal chlorophyll removal rate reaches 87.5% for coatings up to 2 mm thick, while thicker coatings lead to a marked decline in removal efficiency. This suggests a balancing act is necessary between ensuring adequate MOF presence for effective adsorption and avoiding excessive thickness that could impede the interaction between the chlorophyll solution and adsorbent due to reduced membrane porosity.

The UV-visible adsorption spectra of the explored factors affecting adsorption are shown in Figure S2. In essence, these observations underline the importance of optimizing both the solution volume and MOF coating thickness to maximize the degradation performance of the Fe-MOF/non-firing aluminosilicate membranes. These parameters are critical for real-world environmental applications, such as water purification processes, where effective removal of organic contaminants is essential. The data provide a clear guideline, which includes solution volumes within 5 to 15 mL and MOF coating thicknesses at or below 2 mm for efficient chlorophyll adsorption.

In Figure 6f, the UV-Vis adsorption spectra across three cycles of adsorption indicate the recyclability of the Fe-MOF/non-firing aluminosilicate membranes. The membranes show diminishing adsorption capabilities after each cycle, which can be attributed to the partial loss of MOF material during the desorption process with acetone and the blockage of some membrane pores that are too small to be cleaned effectively. The visual change in the chlorophyll solution’s color post-adsorption highlights the membranes’ initial effectiveness in adsorbing chlorophyll.

The reusability of Fe-MOF/non-firing aluminosilicate membranes is a critical factor in their appeal. Unlike single-use adsorbents, these membranes can be regenerated and reused multiple times without a loss in efficiency, which not only makes them more economical over the long term but also aligns with sustainable practices by minimizing waste. Their high separation efficiency ensures that even trace amounts of contaminants are removed, which is crucial for meeting strict regulatory standards in food safety.

The molecular structure of imidacloprid, presented in Figure 6g, illustrates the complexity of effectively adsorbing such molecules from aqueous solutions. The UV-Vis absorption spectra before and after adsorption show that while the composite membrane is capable of selective adsorption, with chlorophyll removal around 90%, a significant portion of imidacloprid remains in the solution. This indicates that while the MOF membrane can selectively adsorb chlorophyll, its effectiveness in adsorbing imidacloprid under the same conditions is limited.

These findings have important implications for the use of such composite membranes in environmental remediation. While the recyclability of the Fe-MOF/non-firing aluminosilicate membranes is a valuable feature, it is evident that their adsorption capacity diminishes with repeated use. Moreover, the selective adsorption properties suggest that while the membranes are effective in removing certain organic compounds, their applicability to other pollutants like neonicotinoid pesticides may require further optimization. Thus, the performance of the reaction system reflects a strong dependency on the physicochemical properties of the pollutants and the structural integrity of the adsorbent after cyclic use.

## 4. Conclusions

In this study, Fe-MOF/non-firing aluminosilicate membranes were successfully explored by optimizing the preparation conditions of the ceramic membranes and the integration of Fe-MOFs. The study effectively employs XRD, SEM, FT-IR, and mechanical testing to explore how these variables influence the ceramic membranes’ physical and chemical attributes, emphasizing the role of sodium hydroxide in enhancing amorphous phases and mechanical strength. This integration results in the membranes not only exhibiting increased mechanical and thermal stability but also enhanced adsorptive properties, making it highly suitable for applications that require both durability and functional performance in environmental and chemical processing industries. The geopolymerization process and the detailed analysis of chemical interactions within the membranes were thoroughly examined, offering valuable insights into tailoring these materials to meet specific industrial needs, ensuring they perform optimally under designated conditions. This study on synthesis parameters and the strategic incorporation of MOFs opens new pathways for the creation of multifunctional membrane materials.

## Figures and Tables

**Table 1:** Non-firing ceramic slurry design table and final membrane materials data.

Sample Number	Al_2_O_3_/SiO_2_ Molar Ratio	NaOH/SiO_2_ Molar Ratio	Sodium Silicate Form	Conservation Methods	Curing Temperature(°C)	BET Surface Area (m^2^/g)	Pore Size (nm)
Y1	1	16:100	Powder	Wet curing	60	28.2	38.1
Y2	1	32:100	Powder	Wet curing	60	1.7	41.8
Y3	1	48:100	Powder	Wet curing	60	0.6	37.3
Y4	1	64:100	Powder	Wet curing	60	0.1	-

## Data Availability

Data are contained within the article.

## Supplementary Materials

The following supporting information can be downloaded at https://www.mdpi.com/article/10.3390/nano14110944/s1, Figure S1: (a,b) Nitrogen adsorption–desorption diagrams for sodium hydroxide/silica molar ratios of 32:100, 48:100, and 64:100; (c) Strength of aluminum silicate non-firing ceramics with different curing times; (d) Strength of aluminum silicate non-firing ceramics with different water contents; Figure S2: UV-visible absorption spectra of (a) Substrates with different sodium hydroxide contents; (b) Different temperatures; (c) Different pH values; (d) Different solution amounts; (e) Different coating thicknesses.
