# Supplementary material for: MOF-Enhanced Aluminosilicate Ceramic Membranes Using Non-Firing Processes for Pesticide Filtration and Phytochrome Removal
Source: Nanomaterials (Basel). 2024 May 27;14(11):944. doi: 10.3390/nano14110944 (PMC11173857; doi:10.3390/nano14110944)
Supplement: Supplementary file 1 [file nanomaterials-14-00944-s001.zip › nanomaterials-2999128-supplementary.pdf]

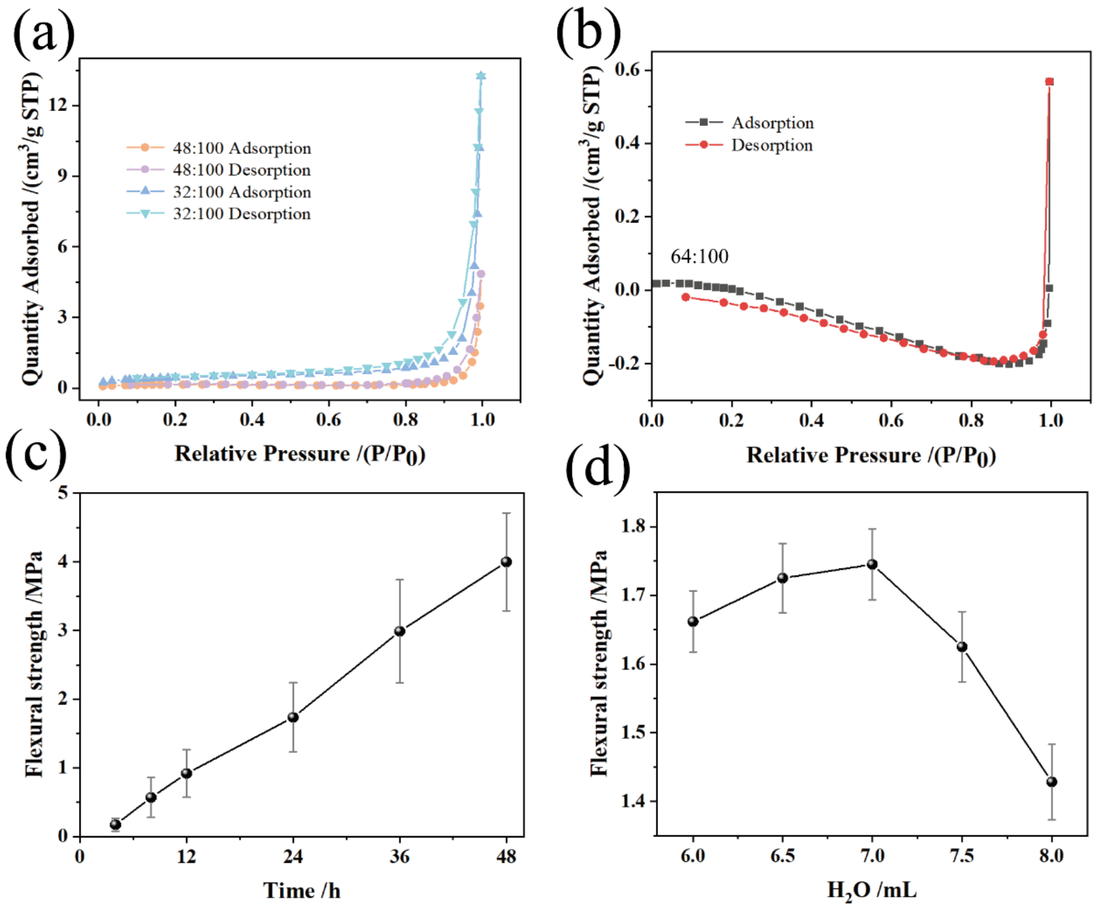

Figure S1. (a) (b) Nitrogen adsorption desorption diagrams for sodium hydroxide to silica molar ratios of 32:100, 48:100, and 64:100; (c) Strength of aluminum silicate non-firing ceramics with different curing times; (d) Strength of aluminum silicate non-firing ceramics with different water contents.

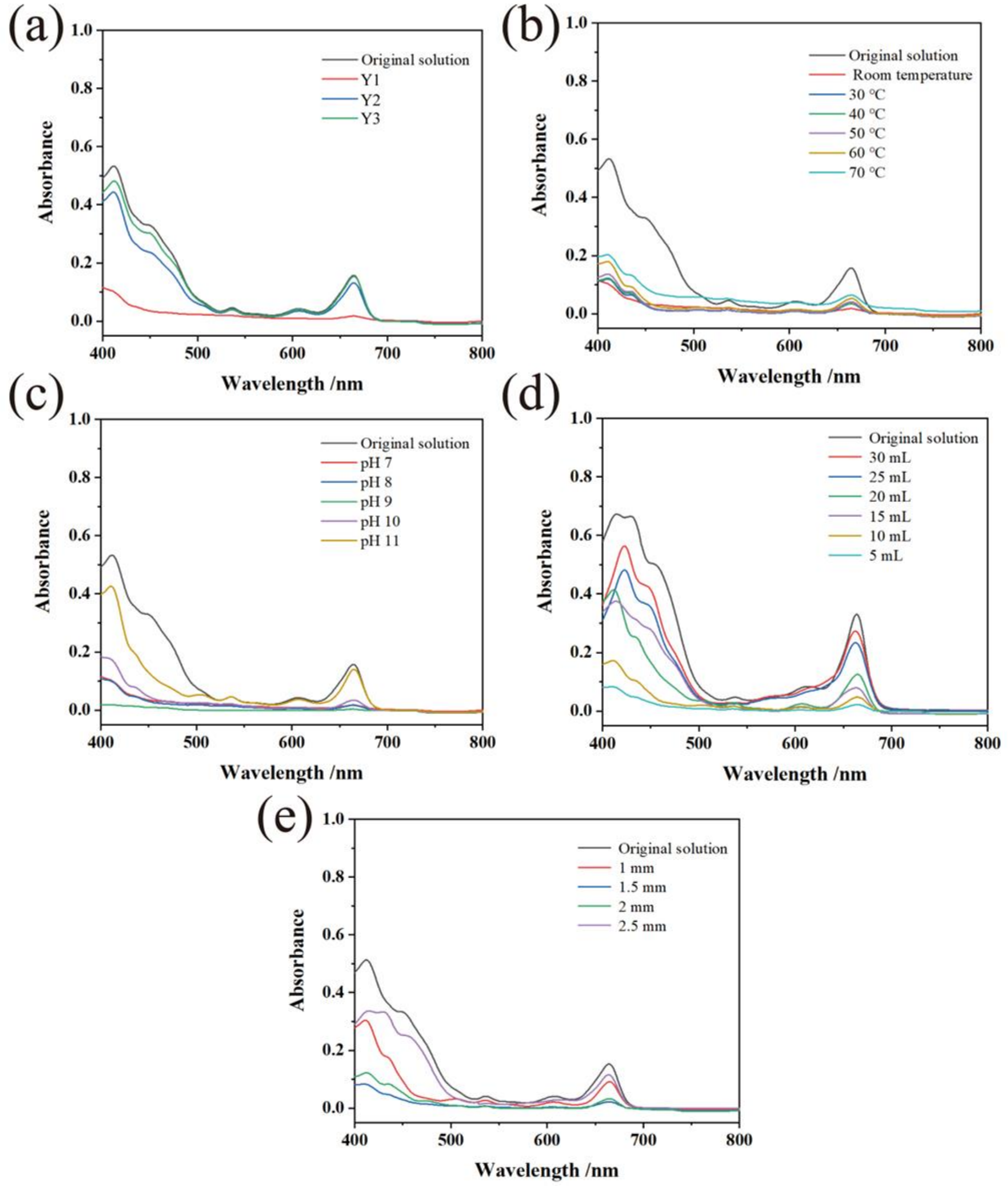

Figure S2. UV-visible absorption spectra of (a) substrates with different sodium hydroxide contents, (b) different temperatures, (c) different pH values, (d) different solution amounts and (e) different coating thicknesses.
